# Tissue‐specific insulin sensitivity in anorexia nervosa: A cross‐sectional positron emission tomography (PET) study in female adults

**DOI:** 10.14814/phy2.71142

**Published:** 2026-10-09

**Authors:** Aino Latva‐Rasku, Kyoungjune Pak, Eleni Rebelos, Miikka‐Juhani Honka, Prince Dadson, Jukka Koffert, Jouni Tuisku, Laura Pekkarinen, Sanna Laurila, Janina Hieta, Kirsi Laitinen, Anna K. Kirjavainen, Jussi Hirvonen, Henry K. Karlsson, Max Karukivi, Lauri Nummenmaa, Pirjo Nuutila

**Affiliations:** ^1^ Turku PET Centre University of Turku and Turku University Hospital Turku Finland; ^2^ Department of Endocrinology Turku University Hospital Turku Finland; ^3^ Department of Nuclear Medicine and Biomedical Research Institute Pusan National University Hospital Busan Republic of Korea; ^4^ School of Medicine Pusan National University Busan Republic of Korea; ^5^ Heart Center Turku University Hospital Turku Finland; ^6^ Institute of Biomedicine & Nutrition and Food Research Center University of Turku Turku Finland; ^7^ Radiopharmaceutical Chemistry Laboratory, Turku PET Centre University of Turku Turku Finland; ^8^ Department of Adolescent Psychiatry Turku University Hospital and University of Turku Turku Finland; ^9^ Department of Psychology University of Turku Turku Finland

**Keywords:** anorexia nervosa, glucose metabolism, growth hormone, positron emission tomography, starvation

## Abstract

Anorexia nervosa (AN) has been linked to both impaired and improved whole‐body insulin sensitivity, but tissue‐specific metabolic alterations remain unexplored. To address this gap, we studied 12 female volunteers with recently diagnosed AN and 13 healthy, lean female controls in a cross‐sectional design using positron emission tomography (PET) imaging with the glucose analogue [^18^F]FDG tracer under hyperinsulinemic conditions. We show that tissue‐specific glucose uptake during hyperinsulinemia was higher only in the duodenum of individuals with AN, but comparable in other tissues, possibly indicating altered intestinal glucose handling and nutrient sensing in reduced energy intake. In five participants with AN, glucose uptake was elevated despite reduced skeletal muscle glucose utilization. In controls, growth hormone (GH) levels correlated with skeletal muscle, liver, and brain insulin sensitivity, but these associations were absent in AN, suggesting disrupted endocrine regulation of glucose metabolism. Overall, whole‐body insulin sensitivity was not altered, yet tissue‐specific glucose metabolism exhibited greater variability in AN. These differences were not directly explained by disease duration or weight loss severity, suggesting contributions from individual genetic background and behavioral patterns.

## Graphical abstract

Anorexia nervosa is associated with increased intestinal glucose uptake and altered ratio of glucose uptake into skeletal muscle versus subcutaneous adipose tissue during systemic hyperinsulinemia.

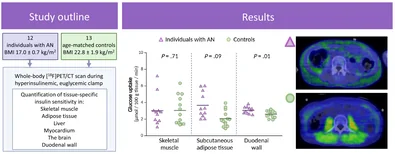

## 1 INTRODUCTION

Anorexia nervosa (AN) is a severe psychiatric disorder characterized by self‐imposed starvation, excessive weight loss, and a distorted body image. The estimated lifetime prevalence of AN is 1.4% in women and 0.2% in men (Galmiche et al., 2019), and it has a high rate of mortality partly due to medical complications associated with the condition (Mehler & Brown, 2015). Recent studies have revealed that individuals with AN experience metabolic changes concerning insulin sensitivity and glucose metabolism in both acute and recovery phases of AN (Haines, 2023; Misra & Klibanski, 2014; Schorr & Miller, 2017; Warren, 2011).

The relationship between insulin sensitivity and anorexia nervosa is multifaceted. In controlled trials using hyperinsulinemic, euglycemic clamp to assess whole‐body insulin sensitivity (DeFronzo et al., 1979), there has been no significant increase in insulin‐stimulated glucose disposal in the acute phase of AN (Castillo et al., 1985; Franssila‐Kallunki et al., 1991; Karczewska‐Kupczewska et al., 2010; Prioletta et al., 2011). In a calorimetry study, glucose oxidation was reported to be upregulated and storing reduced (Franssila‐Kallunki et al., 1991), and this was interpreted to represent diminished skeletal muscle glycogen content in AN. Another study investigating skeletal muscle biopsies from individuals with AN showed, however, that glycogen is more likely accumulated in skeletal muscle due to its inefficient degradation (McLoughlin et al., 1998). During the recovery period insulin sensitivity has been reported to decrease, which is possibly driven by increased visceral adiposity (Kim et al., 2019; Prioletta et al., 2011). Studies using homeostatic model assessment of insulin resistance (HOMA‐IR) as a proxy for insulin sensitivity have reached contrasting conclusions, indicating improved insulin responsiveness in AN (Delporte et al., 2003; Støving et al., 2007; Watson et al., 2019). However, this may be attributed to the typically low fasting glucose and insulin levels observed in AN, which might not accurately reflect heightened responses to insulin.

The relationship between AN and insulin sensitivity likely involves a complex interplay between genetic, epigenetic, biological, psychological, and social factors. For instance, certain genetic factors that reduce the risk of developing obesity or type 2 diabetes are also linked to a higher genetic risk for AN (Duncan et al., 2017; Watson et al., 2019). The hormonal changes in AN, which reflect the body's adaptive responses to severe undernutrition, also result in altered metabolism. In addition to low insulin levels and relative hypercortisolism (Thavaraputta et al., 2023), individuals with AN typically present elevated GH levels (Landon et al., 1966), but they also develop resistance to GH action, resulting in decreased IGF‐1 levels, which can contribute to insulin resistance. Other hormonal changes often reported in AN are reduced leptin levels, elevated ghrelin and peptide YY levels and hypothalamic–pituitary‐gonadal axis dysfunction (Misra & Klibanski, 2014; Schorr & Miller, 2017), but their effects on insulin sensitivity are less well characterized in the context of AN.

These previous findings suggest a redefinition of AN as a metabo‐psychiatric disorder, and therefore elucidating both its metabolic and psychiatric components is a key to improving outcomes and developing novel treatment strategies. Additionally, insulin resistance not only complicates the recovery process but also poses risks for long‐term health outcomes, including the development of metabolic syndrome (Hudson et al., 2020; Zuniga‐Guajardo et al., 1986). However, it is currently not known to what extent AN associates with metabolic changes in different tissues. Therefore, the current study was aimed at exploring the associations between AN and tissue‐specific insulin sensitivity in several different organs by using positron emission tomography (PET) with the glucose analogue radiotracer 2‐deoxy‐2‐[^18^F]fluoro‐D‐glucose ([^18^F]FDG) under hyperinsulinemic, euglycemic clamp conditions. The main aim of the study was to assess differences in brain μ‐opioid receptor availability between individuals with AN and healthy volunteers. These results have been published previously (Pak et al., 2025), whereas the metabolic endpoints presented in this manuscript were experimental, and have not been reported previously.

## 2 MATERIALS AND METHODS

### 2.1 General study outline

The study was a cross‐sectional, observational study to evaluate the metabolic alterations in AN by comparing results from participants with AN and healthy controls. The measures were made on three separate visits: a screening visit, a [^18^F]FDG‐PET/CT scan visit, and a whole‐body MRI scan visit, all performed within 28 days of each other. The study was conducted at the Turku PET Centre, Turku University Hospital, Turku, Finland from August 2020 to March 2023. The study protocol was approved by the Ethics Committee of the Hospital District of Southwest Finland and the study was conducted according to the principles of the Declaration of Helsinki and registered at ClinicalTrials.gov (NCT05101538). All study participants gave written informed consent prior to any study procedures.

### 2.2 Study participants

Participants with AN were recruited by psychiatrists from the Eating Disorder Unit at the Turku University Hospital, whereas the control participants were recruited via university and hospital internet page message boards.

Only female participants were recruited due to the significantly higher prevalence of AN in women, as sex could have interfered with the results in this small sample. All participants with AN were to meet the following criteria: (1) modified DSM‐IV diagnosis of AN with or without amenorrhea; (2) AN onset before the age of 25 years and not more than 2 years prior to screening; (3) age 18–30 years; (4) BMI less than 17.5 kg/m^2^; (5) no lifetime history of binge eating. Due to difficulties in the recruitment process, longer disease duration was, however, allowed. In the control group the participants were to be age‐matched to the AN participants, and to have no reported history of eating disorders or obesity (BMI over 30 kg/m^2^), first degree relatives with an eating disorder, or psychiatric disorders. All participants were female, had no use of tobacco or narcotics, had no significant previous exposure to radiation, and were required to use reliable contraception during the study.

### 2.3 Screening visit

On the screening visit, concomitant medications and medical history were recorded, and the participants underwent a physical examination. Fasting blood samples were collected, followed by a 75 g oral glucose tolerance test (OGTT). Body fat mass and fat‐free mass were quantified with air displacement plethysmograph (BOD POD, version 5.4.0, COSMED Inc., CA, USA) after a minimum of 4 h of fasting.

### 2.4 PET study visit protocol

The study visit was performed after an overnight fast. First, two venous catheters were inserted in opposite forearms, one for the insulin and glucose infusions and for injecting [^18^F]FDG, and the other for collecting venous blood samples, arterialised by placing a hot water bottle distally on the arm. After the collection of fasting plasma blood samples, hyperinsulinemic, euglycemic clamp was started (DeFronzo et al., 1979). Insulin (Actrapid, Novo Nordisk A/S, Bagsvaerd, Denmark) was administered with a reduced dose of 37 mU/m^2^ body surface area/min, and a variable rate of 20% glucose was infused based on plasma glucose measurements performed every 5–10 min to maintain euglycemia (plasma glucose 5.0 mmol/L).

The participants were then transferred to a combined PET/CT scanner (Discovery 690 or Discovery MI; General Electric Medical Systems, Milwaukee, WI), and 75 min (SD 11) into the clamp, 150 MBq (SD 7) of [^18^F]FDG was administered together with the start of dynamic scans of the thoracic region (32 min) and the brain (15 min), followed by static frames of the neck (10 min), abdomen (10 min), and the thighs (10 min). Next, the participants were transferred from the scanner; insulin and glucose infusions were ceased, and the study participants had a meal and were monitored until the plasma glucose levels were adequate before being discharged.

### 2.5 PET data analysis

Before analysis, all PET data was corrected for dead time, decay, and photon attenuation. Input function was obtained by combining activity data from PET images from inside the left ventricle, and plasma samples collected at 8–11 time points during the scan and analyzed with an automated gamma counter (Wizard 1480 3, Wallac, Turku, Finland). Tissue radioactivity was determined using Carimas software (Version 2.9, Turku PET Centre) (Rainio et al., 2023), by manually applying 3D volumes of interest to the right lobe of the liver, quadriceps femoris muscles, left ventricular myocardium, waistline and mid‐thigh subcutaneous adipose tissue, supraclavicular brown adipose tissue, humeral shaft bone marrow and in the descending part of duodenal wall. Tissue tracer uptake rate was then calculated with graphical analysis (K_i_ or fractional uptake rate [FUR]) by comparing tissue radioactivity accumulation rate to the integral of plasma radioactivity (Patlak & Blasberg, 1985; Thie, 1995).

Tissue glucose uptake (GU, μmol/100 g/min) was then calculated by multiplying K_i_ or FUR with the mean plasma glucose level during the scan, and dividing it by tissue density and a constant accounting for the differences in the metabolic rates of [^18^F]FDG and glucose (1.2 for skeletal muscle (Peltoniemi et al., 2000), 1.0 for liver (Iozzo et al., 2007), 1.14 for adipose tissue (Virtanen et al., 2001), 0.65 for the brain (Wu et al., 2003)).

Glucose uptake in the brain was obtained using SPM12‐based toolbox Magia (Karjalainen et al., 2020), with the data spatially normalized to MNI space using an in‐house [^18^F]FDG template and finally smoothed with a Gaussian kernel with 8‐mm full‐width at half‐maximum.

EGP was assessed by subtracting the rate of glucose infused during the [^18^F]FDG‐PET/CT study from the whole‐body glucose disappearance rate (*R*_d_) derived from [^18^F]FDG consumption (Iozzo et al., 2006).

### 2.6 Whole‐body MRI

A whole‐body MRI scan was performed at 3 T using the MRI part of a clinical PET‐MR system (Philips Ingenuity TF, Philips Healthcare, OH, USA) using a 2‐point Dixon sequence (LAVA Flex 3D, flip angle 12°, echo time 1.7 ms, repetition time 4.4 ms, slice thickness 1.5 mm). Volumes of visceral and subcutaneous adipose tissues were defined manually from Th12‐L1 to L5‐S1 level using sliceOmatic (version 5.0, Tomovision, Canada). Tissue fat fractions (FF%) were analyzed from maps calculated using in‐house software developed in MATLAB2015b (Mathworks Inc., Natick, MA) and SPM8 (Wellcome Trust, UCL) using the formula fat fraction = fat component / (fat component + water component). Using Carimas software (version 2.9, Turku PET Centre), 3D volumes were drawn similarly to PET data analyses, with the addition of visceral adipose tissue volumes of interest being drawn in lower retroperitoneal space.

### 2.7 Laboratory analyses

On the screening visit during OGTT, glucose and insulin were measured every 30 min for 2 h. Cholesterols, thyroid stimulating hormone (TSH), free T4, and T3 were also measured at screening, whereas other laboratory samples were analyzed from samples collected during the PET visit. During clamp, plasma glucose was measured in duplicates using the glucose oxidase method. Plasma insulin was determined every 30 min, and serum free fatty acids (FFA) and growth hormone (GH) were measured every 60 min during the clamp. Plasma radioactivity was determined every 5–15 min using an automatic gamma counter, and the amount of [^18^F]FDG lost into urine was measured from a urine sample given at the end of the scan using an isotope dose calibrator.

During clamp, plasma glucose was measured in duplicates using the glucose oxidase method (Analox GM9 Analox Instruments, London, UK). Plasma insulin, TSH, free T4 and T3 were assessed using an automated electrochemiluminescence immunoassays (ECLIA, Roche Diagnostics, Mannheim, Germany), serum free fatty acid (FFA) levels with an enzymatic colorimetric method assay (NEFA‐HR2, ACS‐ACOD, Wako Chemicals, Neuss, Germany; Cobas 8000 c502 Analyzer, Roche Diagnostics GmbH, Mannheim, Germany), cholesterols were measured using an enzymatic photometric assay (Roche Diagnostics, Mannheim, Germany). Serum GH was assessed in ISLAB, Kuopio, Finland using a chemiluminescent immunoassay (CLIA, LIAISON XL, DiaSorin S.p.A., Saluggia, Italy).

### 2.8 Statistical analyses

The sample size was set to detect a significant difference between individuals with AN and controls in μ opioid receptor availability, reported separately (Pak et al., 2025), and no separate power calculations for metabolic endpoints were determined. The study was initially planned to recruit 30 individuals with AN and 30 control participants. However, recruitment was slower than anticipated, and 12 patients and 13 controls were ultimately included. During recruitment, the primary endpoint reached statistical significance. Considering the observed result, slow recruitment, and the additional exposure to ionizing radiation associated with the study procedures, recruitment was discontinued to avoid unnecessary participant burden and radiation exposure. The smaller‐than‐planned sample size is acknowledged as a limitation of the study.

Normality of the data was evaluated with the Shapiro–Wilk test. Square root or logarithmic transformations were performed for non‐normally distributed data. Differences between groups were compared using independent samples *t*‐test, except for the screening visit HDL cholesterol and TSH analyzed with the Mann–Whitney *U* test. Associations were studied with Pearson correlation. All statistical analyses were performed using IBM SPSS Statistics for Windows (version 29.0, IBM Corp, Armonk, NY, USA).

## 3 RESULTS

### 3.1 Participant characteristics

Participant characteristics have been reported previously (Pak et al., 2025). Briefly, the cohort comprised 12 individuals with AN and 13 healthy controls after one participant with AN withdrew from the study before the [^18^F]FDG study. Individuals with AN were younger than controls (20.6 [SD 1.7] vs. 23.9 [SD 3.2] years, *p* = 0.005) and had lower BMI (17.0 [SD 0.7] vs. 22.8 [SD 1.9] kg/m^2^, *p* < 0.001), body weight (48.3 [SD 3.8] vs. 62.6 [SD 5.0] kg, *p* < 0.001), and body fat percentage (19.6 [SD 7.5] % vs. 33.6 [SD 5.4] %, *p* < 0.001). Fasting plasma glucose was also lower in individuals with AN (4.7 [SD 0.3] vs. 5.0 [SD 0.4] mmol/L, *p* = 0.03). Total cholesterol (3.8 [SD 0.7] vs. 4.3 [SD 0.7] mmol/L, *p* = 0.13), LDL cholesterol (2.1 [SD 0.7] vs. 2.5 [SD 0.4] mmol/L, *p* = 0.17), HDL cholesterol (1.8 [SD 0.7] vs. 1.7 [SD 0.3] mmol/L, *p* = 0.89), and triglycerides (0.6 [SD 0.3] vs. 0.9 [SD 0.5] mmol/L, *p* = 0.07) did not differ significantly between groups. Previously unpublished characteristics of the two study groups are presented in Table 1. The duration of eating disorders in the AN group ranged from 1 to 11 years (mean 4.3 [SD 3.1] years), and 9/11 of individuals with AN had amenorrhea.

**TABLE 1:** Participant characteristics.

	Individuals with AN (*N* = 12)	Controls (*N* = 13)	*p* value
Fat mass (kg)	9.6 ± 4.3	21.1 ± 4.6	<0.001
Lean mass (kg)	38.6 ± 2.6	41.5 ± 3.5	0.03
WHR	0.75 ± 0.05	0.74 ± 0.05	0.73
Fasting plasma insulin (pmoL/L)	26.4 ± 10.4	54.2 ± 26.0	0.002
Fasting free fatty acids (mmol/L)	0.54 ± 0.31	0.65 ± 0.18	0.11
Fasting β‐hydroxybutyrate (mmol/L)	0.18 ± 0.13	0.16 ± 0.08	0.61
HOMA‐IR	0.8 ± 0.3	1.7 ± 1.0	0.003
Matsuda‐ISI	9.6 ± 3.6	5.5 ± 1.9	0.001
TSH (mIU/L)	1.9 ± 0.6	2.0 ± 1.8	0.32
Free T4 (pmol/L)	13.4 ± 1.9	14.6 ± 1.5	0.08
Free T3 (pmol/L)	3.7 ± 0.6	5.0 ± 0.9	<0.001
IGF‐1 (nmol/L)	28.0 ± 8.2	28.4 ± 8.8	0.91
Fasting GH (μg/L)	2.7 ± 1.6	2.4 ± 2.9	0.11
GH during HEC (μg/L)	2.3 ± 1.9	1.0 ± 1.4	0.02
Cortisol (nmol/L)	261 ± 76	356 ± 250	0.21
Leptin (μg/L)	2.3 ± 2.0	13.7 ± 7.0	<0.001

*Note*: The data are means and SDs, and the *p* values are from *t*‐test comparisons between groups.

Abbreviations: GH, growth hormone; HEC, hyperinsulinemic, euglycemic clamp; IGF‐1, insulin‐like growth factor 1; OGTT, oral glucose tolerance test; TSH, thyroid stimulating hormone; WHR, Waist‐hip‐ratio.

### 3.2 Whole‐body and tissue glucose metabolism in AN

During the steady state of clamp, mean glucose (5.0 [SD 0.3] mmol/L vs. 5.0 [SD 0.2] mmol/L, *p* = 0.50) and insulin levels (442.7 [SD 90.0] pmol/L vs. 417.4 [SD 58.1] pmol/L, *p* = 0.42) were similar in individuals with AN and healthy controls, respectively. After 60 min of clamp, serum FFA levels were slightly lower in individuals with AN (0.04 [SD 0.02] mmol/L vs. 0.06 [SD 0.03] mmol/L, *p* = 0.04), but the levels were more similar between the groups at fasting (Table 1), and 120 min into the clamp. Also, the change from fasting to 60 min of hyperinsulinemia was equal between the groups (−0.50 [SD 0.30] mmol/L in individuals with AN vs. −0.59 [SD 0.16] mmol/L in controls, *p* = 0.38).

Levels of fasting insulin, cortisol, TSH, free T4, free T3, IGF‐1, or leptin did not correlate with peripheral measures of insulin sensitivity in the whole study group or separately in participants with AN (*p* values >0.20).

Whole‐body insulin‐stimulated glucose disposal measured as *M* value was higher in participants with AN when using total body weight, but not fat free mass, as a reference. Rate of endogenous glucose production (EGP) during hyperinsulinemic euglycemia was not different between groups (Figure 1a; Table S1). Tissue‐specific glucose uptake was statistically significantly higher only in the duodenal wall (3.0 [SD 0.4] μmol/100 g/min in the participants with AN vs. 2.6 [SD 0.4] μmol/100 g/min in controls, *p* = 0.013) (Figure 1b; Table S1).

**FIGURE 1:**
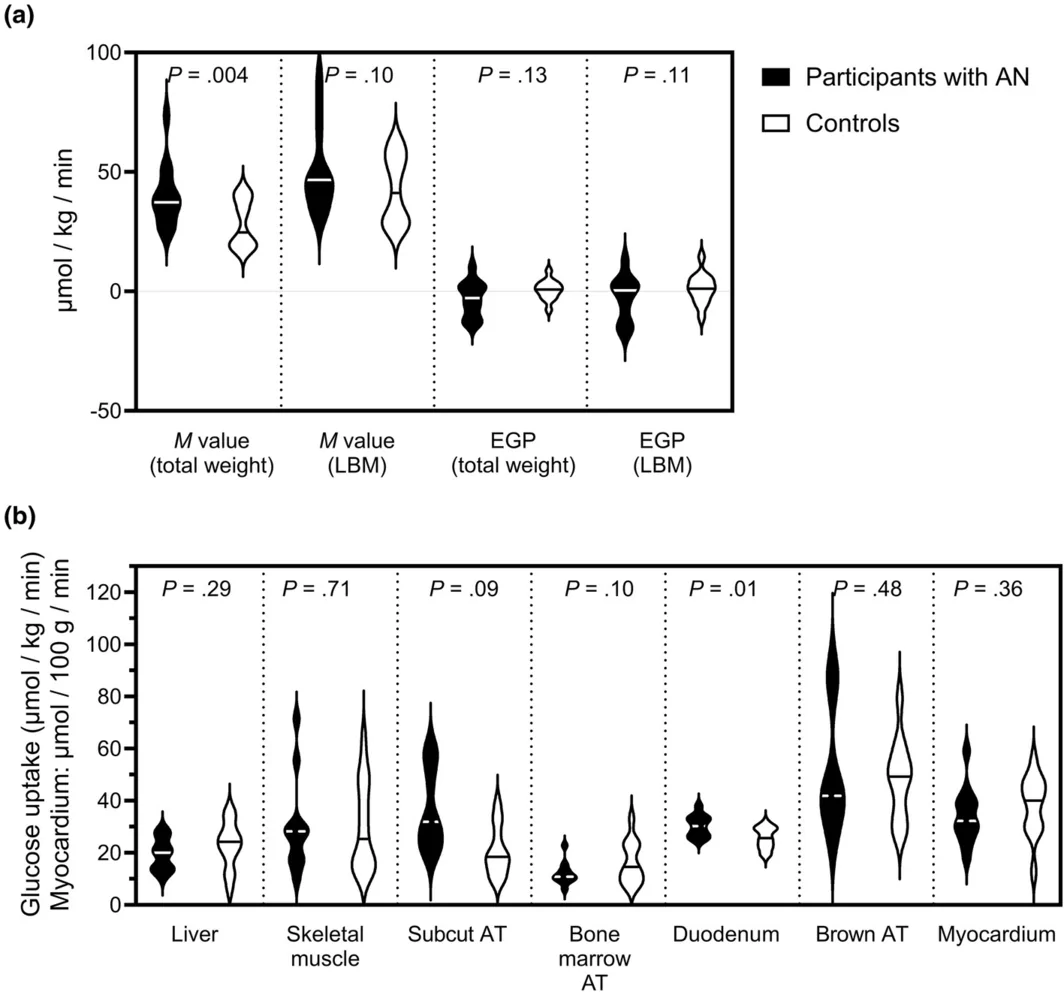
(a) *M* value was higher in the participants in AN when using total body weight, but not lean body mass (LBM) as reference. Endogenous glucose production (EGP) during hyperinsulinemia did not differ between groups. (b) Insulin‐stimulated glucose uptake was statistically higher only in the duodenal wall in participants with AN. AT, adipose tissue. Violin plots illustrate the distribution of observations within each group. Horizontal lines denote group means. Mean (SD) values are reported in the text.

There was a negative correlation with the duration of AN and glucose uptake in the duodenal wall (*r* = −0.64, *p* = 0.04), but not with other tissues (*p* values above 0.41).

There were no significant differences between individuals with AN and controls in brain glucose uptake rates during hyperinsulinemia in the whole brain (23.0 [SD 4.3] μmol/100 g/min vs. 24.6 [SD 2.5] μmol/100 g/min, respectively, *p* = 0.30), white matter (19.6 [SD 3.8] μmol/100 g/min vs. 20.8 [SD 2.1] μmol/100 g/min, respectively, *p* = 0.36), or gray matter (26.9 [SD 5.2] μmol/100 g/min vs. 28.6 [SD 3.2] μmol/100 g/min, respectively, *p* = 0.36), or in specific anatomical brain regions.

### 3.3 Varying tissue insulin‐stimulated glucose uptake in skeletal muscle and adipose tissue

Although glucose uptake to subcutaneous adipose tissue (AT) was numerically slightly higher in participants with AN, uptake rate in the total subcutaneous AT depot was higher in the control participants (44.2 [SD 15.3] μmol/min vs. 29.2 [SD 16.6] in participants with AN, *p* = 0.05). Based on post hoc analyses performed after visually evaluating the PET data, five participants with AN, glucose uptake rate was higher in subcutaneous AT (5.0 [SD 1.0] μmol/100 g/min) than in skeletal muscle (1.8 [SD 0.6] μmol/100 g/min) (Figure 2). This was mostly driven by increased subcutaneous AT glucose uptake, while the difference in skeletal muscle GU was only lower compared to other participants with AN (4.0 [SD 1.9] μmol/100 g/min, *p* = 0.04) and not with healthy controls (3.0 [SD 1.7] μmol/100 g/min, *p* = 0.17). Glucose uptake to the total waistline subcutaneous adipose tissue depot was also numerically higher in the five participants (46.2 [SD 8.4] μmol/min vs. 34.7 [SD 23.1] μmol/min in other participants with AN and 35.2 [SD 17.0] μmol/min in controls), but the differences were not statistically significant (*p* = 0.29 and *p* = 0.24, respectively). In the whole study group, mean insulin levels during the clamp correlated negatively with subcutaneous adipose tissue GU (*r* = −0.76, *p* = 0.001).

**FIGURE 2:**
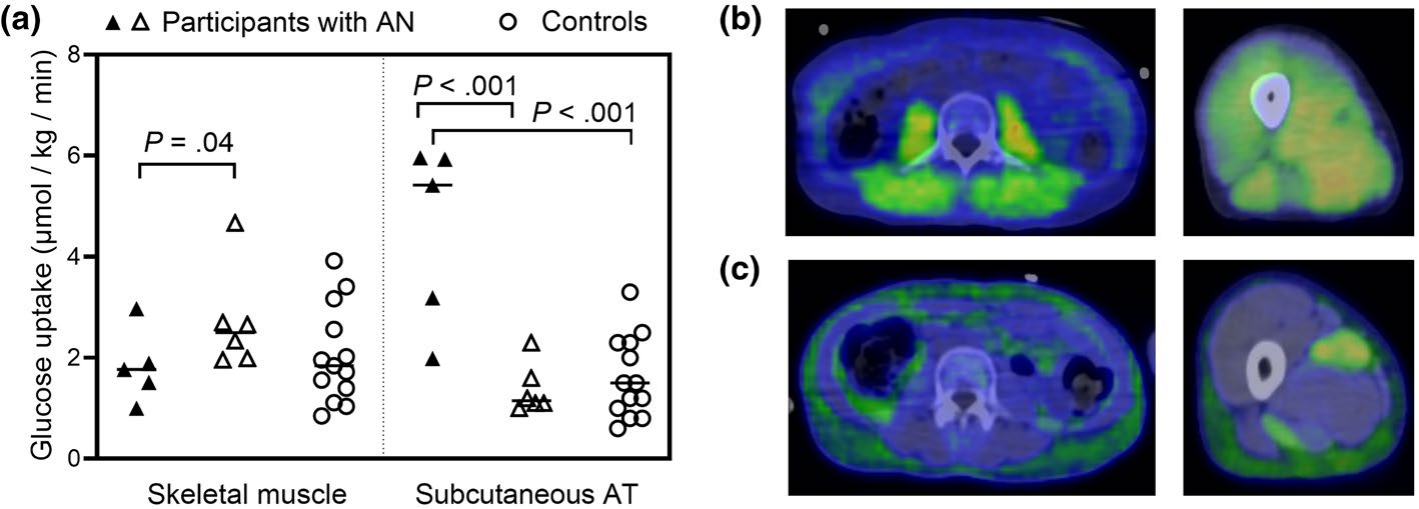
(a) In five participants with AN, the rate of glucose uptake was increased in subcutaneous adipose tissue in comparison to skeletal muscle (black triangles), mostly driven by increased adipose tissue glucose uptake, as skeletal muscle glucose uptake was only lower compared to other participants with AN (white triangles) but not healthy controls (*p* = 0.17, circles). (b, c) [^18^F]FDG‐PET/CT images from a waistline transverse section (left) and from right mid‐thigh (right), with B showing a typical pattern of insulin‐stimulated glucose uptake and C increased glucose uptake to adipose tissue compared to skeletal muscle.

### 3.4 The relationship with GH and tissue glucose metabolism

There was no difference in fasting GH levels between groups, but 60 min into hyperinsulinemic, euglycemic clamp GH remained at a higher level in individuals with AN (Table 1). One healthy control had exceptionally high GH levels both in fasting conditions (9.5 μg/L) and during hyperinsulinemia (4.9 μg/L), but was included in the analyses.

In the normal‐weight control group, higher GH during hyperinsulinemic euglycemia was associated with impaired insulin sensitivity. GH correlated negatively with *M* value (*r* = −0.87, *p* < 0.001) and skeletal muscle glucose uptake (*r* = −0.76, *p* = 0.006), and positively with EGP (*r* = 0.64, *p =* 0.02) and brain glucose uptake (*r* = 0.79, *p* = 0.004). These associations were absent in individuals with AN (*p* values ≥0.37) (Figure 3). In the whole group, fasting GH levels correlated negatively with free fatty acid levels (*r* = −0.48, *p* = 0.02), whereas during hyperinsulinemic clamp the correlation was absent (*r =* 0.01, *p* = 0.96) also when looking at the groups separately (*p* values >0.60).

**FIGURE 3:**
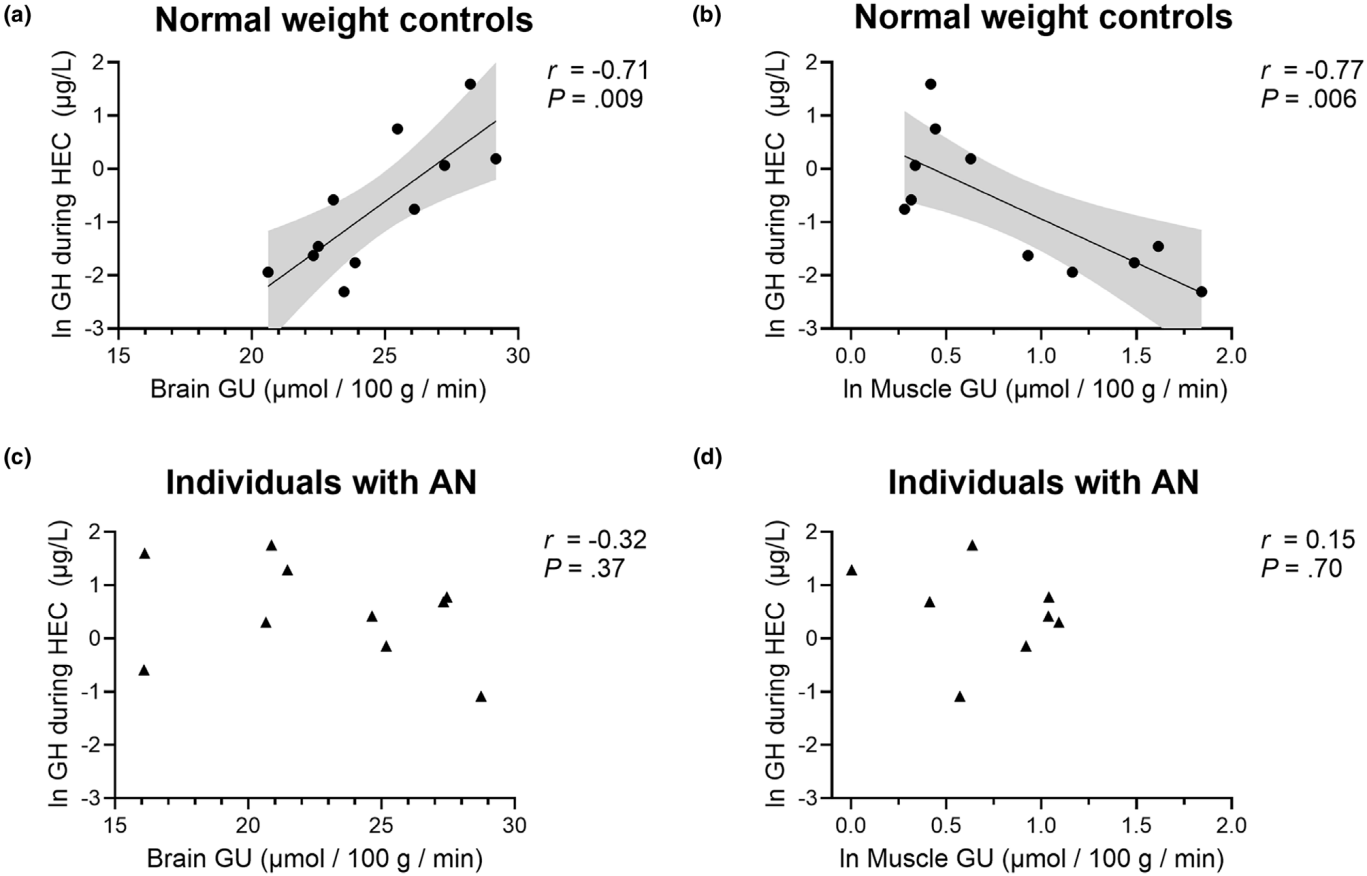
During hyperinsulinemic euglycaemia, higher growth hormone (GH) levels correlated with typical characteristics of insulin resistance, such as elevated brain glucose metabolism and lower skeletal muscle glucose uptake, in the normal weight controls (a, b), but not in individuals with AN (c, d). GU, glucose uptake. GH and skeletal muscle GU values are ln transformed to reach normal distribution.

### 3.5 Different profiles in adipose tissue fat fractions

Participants with AN had significantly lower volumes of abdominal and femoral subcutaneous adipose tissue, visceral adipose tissue, and femoral skeletal muscle (Table 2). Tissue proton density fat fractions were similar in the liver, skeletal muscle, and visceral adipose tissue. While adipose tissue fat fraction was lower in the subcutaneous abdominal and femoral depots in individuals with AN, it was higher in the femoral bone marrow (Table 2).

**TABLE 2:** Tissue volumes and fat fractions.

	Individuals with AN	Controls	*p* value
Tissue volumes (mL)			
Abdominal subcutaneous AT	776 ± 289	2321 ± 787	<0.001
Femoral subcutaneous AT	2023 ± 555	4560 ± 1243	<0.001
Visceral AT	110 ± 60	404 ± 297	<0.001
Thigh skeletal muscle	3426 ± 580	4185 ± 660	0.003
Proton‐density fat fractions (%)			
Abdominal subcutaneous AT	80.5 ± 8.9	90.4 ± 3.8	0.01
Femoral subcutaneous AT	83.8 ± 8.9	90.3 ± 3.4	0.03
Visceral AT	84.7 ± 6.5	87.7 ± 4.1	0.18
Femoral bone marrow AT	94.0 ± 2.5	91.0 ± 3.1	0.02
Brown AT	79.8 ± 6.8	76.9 ± 8.9	0.39
Thigh skeletal muscle	4.8 ± 2.5	4.3 ± 2.2	0.65
Liver	3.8 ± 1.4	3.7 ± 1.2	0.87

*Note*: The data are means and SDs, and the *p* values are from *t*‐test comparisons between groups.

Abbreviation: AT, adipose tissue.

## 4 DISCUSSION

This is the first study to investigate the association between AN and glucose metabolism in several different tissues within the same individuals. The results on the whole‐body level are in line with several previous reports, but this study also improves our understanding of the altered glucose homeostasis and organ crosstalk in AN, with special emphasis on adipose tissue, skeletal muscle, and the brain.

Previous studies using hyperinsulinemic, euglycemic clamp have shown that AN is not associated with improved insulin sensitivity (Castillo et al., 1985; Franssila‐Kallunki et al., 1991; Karczewska‐Kupczewska et al., 2010; Prioletta et al., 2011). As skeletal muscle is the main site of glucose uptake during hyperinsulinemia, uptake measured with [^18^F]FDG‐PET was expected to show no difference between individuals with AN and normal‐weight controls.

In five individuals with AN, insulin‐stimulated glucose uptake into skeletal muscle was impaired compared to subcutaneous adipose tissue, which is in contrast to the rest of the study population and a larger dataset of 326 individuals studied earlier in Turku PET Centre (Honka et al., 2018). This could not be directly explained by differences in levels of circulating substrates, but there was an association between higher adipose tissue GU and lower plasma insulin levels during the clamp, which can be interpreted as higher insulin clearance. This is in line with previous data, as prolonged fasting has been reported to induce insulin resistance in skeletal muscle (Blackwood et al., 2023; Newman & Brodows, 1983), and to potentiate insulin binding to adipocytes in vitro (Kolterman et al., 1979). Also, in a rat model repeated cycles of starvation and refeeding result in significant upregulation of white adipose tissue lipogenesis (Silva et al., 2019), which would require higher glucose supply to the tissue (Kochan et al., 1997). Therefore, it is possible that the individuals with higher adipose tissue glucose uptake had a longer period of low‐calorie intake than others, have a history of repeating starvation‐refeeding periods, or they might for example, express starvation myopathy or a different profile on myocyte fiber types (McLoughlin et al., 1998). Also, both skeletal muscle insulin resistance and increased subcutaneous AT glucose uptake can be caused or incremented by hypercortisolism (Geer et al., 2014; Kolterman et al., 1979) and elevated GH levels (Tavares et al., 2022), which are common findings in AN. While we did not observe differences in fasting cortisol levels between individuals with AN and controls, this can be attributed to the use of oral contraceptives in the control group.

For other AT depots, the increase in bone marrow fat content has been reported by several groups previously and suggested to be associated with impaired bone health in AN (Bredella et al., 2009). Also, in line with previous studies, we observed a more significant reduction in subcutaneous AT volumes but a lesser difference in the volume of visceral AT and no difference in visceral AT fat fraction (Mayo‐Smith et al., 1989). Young females with AN have previously been shown to express less cold‐activated brown AT than healthy controls (Bredella et al., 2012), and this is not in disagreement with our studies as cold activation remains the only method to reliably detect brown AT using PET imaging.

Changes in intestinal metabolism in anorexia have not been reported earlier, but gastrointestinal complications, such as delayed gastric emptying, delayed small bowel transit time, or constipation, are common in individuals with AN (Mehler & Brown, 2015). In animal models, extended fasting has been associated with increased glucose transporter expression on the luminal surface of enterocytes (Koepsell, 2020; Nakamura et al., 2023), but the uptake of D‐glucose from circulation has gained even less attention. As a result, we can only speculate that chronic undernourishment in AN results in upregulated nutrient uptake from the circulation, and note that due to the small sample size the current finding must be considered exploratory. Lower duodenal glucose uptake in the individuals with longer disease duration might be explained by a reduction in mucosal surface area as seen in chronic malnutrition (Tamlyn & Das, 2021), but this cannot be confirmed from the data collected in the current study. The increased duodenal glucose uptake observed in the present study also raises the possibility that altered enteroendocrine activity contributes to metabolic adaptation in AN. In particular, incretin hormones such as GLP‐1 and GIP may represent mechanistic links between intestinal nutrient sensing, central appetite regulation, and peripheral insulin sensitivity (Kolb et al., 2026).

Brain glucose metabolism in AN has previously been studied only in fasting conditions. Delvenne et al. reported globally lowered cerebral glucose uptake in the acute phase, and this change was reversed after weight restoration (Delvenne et al., 1996). Other studies using predetermined rate constants or measuring relative changes in the brain have reported regional hyper‐ or hypometabolism, but due to methodological differences, the results from these studies are difficult to compare (Herholz et al., 1987; Zhang et al., 2013). As we saw no significant differences between groups, the increased availability of free fatty acids or ketone bodies may compete with glucose uptake in the fasted state, and as lipolysis and ketone production are suppressed during hyperinsulinemia, these differences in glucose uptake also diminish. However, we also noted hypometabolism in some of the individuals with AN; and second, as the participants in the control group were slightly older and aging has been shown to reduce brain glucose uptake under the hyperinsulinemic, euglycemic condition (Rebelos et al., 2021), it is possible that the current small observational study lacked the power to detect a difference.

Similarly to previous studies using oral or intravenous glucose tolerance tests to study hormonal responses in AN (Misra et al., 2004; Tamai et al., 1991), in our study the growth hormone level remained elevated in individuals with AN after 60 min of hyperinsulinemic euglycemia. The lost association between GH levels and skeletal muscle insulin‐stimulated glucose uptake, endogenous glucose production during hyperinsulinemia, and, as a result, whole‐body insulin sensitivity in individuals with AN can be interpreted to associate with the previously established GH resistance and the disrupted pulsatile secretion of GH in individuals with AN (Misra & Klibanski, 2014; Støving et al., 1999). Considering the association between brain glucose uptake and GH, we have previously shown in a larger dataset that obesity and insulin resistance are associated with increased brain glucose uptake during hyperinsulinemia (Rebelos et al., 2021). The higher GH levels and increased brain glucose uptake observed during hyperinsulinemia might both result from peripheral or hepatic insulin resistance. However, it is also possible that an increased cerebral demand for glucose stimulates GH secretion, which in turn induces peripheral insulin resistance to ensure sufficient glucose availability for the brain (Tavares et al., 2022)—and that this regulatory system is disrupted in AN.

While this study offers exciting insights into metabolic alterations in AN, the results are limited by the small sample size. This also prevents us from being able to differentiate between educational level or varying behavioral patterns in AN, with restricted feeding or excessive physical exercise possibly explaining some of the variations within the AN group. Due to the cross‐sectional approach, we cannot determine a causal relationship between the hormonal changes and tissue glucose metabolism. Also, for the interpretation of the GH results, collecting samples for ghrelin measurement could have been of interest. Finally, as we only studied women due to the higher prevalence of AN in this sex, the results may not be generalized to men.

To conclude, in our small cohort AN was not associated with improved insulin sensitivity, but the results provide foundation for further investigation into the interplay between glucose metabolism, hormonal regulation, and organ crosstalk. It is likely that different genetic, epigenetic, behavioral, and hormonal factors can cause a varying metabolic phenotype, with some individuals with AN showing heightened and others impaired insulin sensitivity in skeletal muscle and subcutaneous adipose tissue. Of all the hormonal and metabolic factors studied, the level of growth hormone during steady hyperinsulinemic euglycemia – and its association with glucose metabolism in skeletal muscle, the liver, and the brain – was the most distinctive difference between individuals with AN and healthy controls. These insights may ultimately inform more targeted approaches to managing the metabolic consequences of AN.

## AUTHOR CONTRIBUTIONS

**Aino Latva‐Rasku:** Conceptualization; formal analysis; investigation; methodology; project administration; visualization. **Kyoungjune Pak:** Conceptualization; formal analysis; investigation. **Eleni Rebelos:** Conceptualization; formal analysis; investigation. **Miikka‐Juhani Honka:** Data curation; formal analysis. **Prince Dadson:** Data curation; formal analysis. **Jukka Koffert:** Data curation; formal analysis. **Jouni Tuisku:** Data curation; formal analysis; visualization. **Laura Pekkarinen:** Data curation; investigation. **Sanna Laurila:** Conceptualization; investigation. **Janina Hieta:** Data curation; investigation. **Kirsi Laitinen:** Data curation; investigation. **Anna K. Kirjavainen:** Data curation; investigation. **Jussi Hirvonen:** Data curation; investigation. **Henry K. Karlsson:** Conceptualization; investigation. **Max Karukivi:** Conceptualization; supervision. **Lauri Nummenmaa:** Conceptualization; funding acquisition; project administration; resources; supervision. **Pirjo Nuutila:** Conceptualization; funding acquisition; project administration; resources; supervision.

## FUNDING INFORMATION

This study was supported by Finnish Medical Foundation (A.L.‐R. and S.L.), State Research Funding, The wellbeing services county of Southwest Finland (A.L‐R. and P.D.), Sigrid Juselius Foundation (L.N.), Gyllenberg's stiftelse (L.N.) and Research Council of Finland (L.N.: 294897 and 332225).

## CONFLICT OF INTEREST STATEMENT

The authors declare no conflicts of interest.

## ETHICS STATEMENT

The study protocol was approved by the Ethics Committee of the Hospital District of Southwest Finland (Case no 127/1801/2019) and the study was conducted according to the principles of the Declaration of Helsinki.

## Supporting information


**Table S1.** Whole‐body glucose uptake rates, endogenous glucose production rate and insulin‐stimulated glucose uptake rates in peripheral tissues.

## Data Availability

Source data for this study is not publicly available due to privacy or ethical restrictions. The source data are available to verified researchers upon request by contacting the corresponding author.
