# Supplementary material for: Tissue‐specific insulin sensitivity in anorexia nervosa: A cross‐sectional positron emission tomography (PET) study in female adults
Source: Physiol Rep. 2026 Oct 9;14(19):e71142. doi: 10.14814/phy2.71142 (PMC13653040; doi:10.14814/phy2.71142)
Supplement: Supplementary file 1 [file PHY2-14-e71142-s001.docx]

*Latva-Rasku et al.*

**Tissue-specific insulin sensitivity in anorexia nervosa, a cross-sectional positron emission tomography study**

**Online-only supplemental material**

### ***Supplemental table 1. Whole-body glucose uptake rates, endogenous glucose production rate and insulin-stimulated glucose uptake rates in peripheral tissues***

|  | Participants with AN (*N* = 12) | Controls  (*N* = 13) | *P* value |
| --- | --- | --- | --- |
| *M* value (µmol/kg/min) | 40.2 ± 13.4 | 28.4 ± 10.0 | .004 |
| *M* value per lean mass (µmol/kg_FFM_/min) | 50.5 ± 18.1 | 42.7 ± 14.4 | .10 |
| EGP (µmol/kg/min) | -3.3 ± 7.6 | 0.6 ± 4.1 | .12 |
| EGP per lean mass (µmol/kg_FFM_/min) | -4.4 ± 9.9 | 1.1 ± 6.4 | .11 |
| Tissue glucose uptake rates (µmol/100 g/min) | | | |
| Myocardium | 34.1 ± 10.7 | 38.2 ± 11.5 | .36 |
| Liver | 2.0 ± 0.6 | 2.3 ± 0.9 | .29 |
| Skeletal muscle | 3.0 ± 1.8 | 3.0 ± 1.7 | .71 |
| Duodenum | 3.0 ± 0.4 | 2.6 ± 0.4 | .013 |
| Waistline subcutaneous AT | 2.7 ± 2.3 | 1.4 ± 1.0 | .09 |
| Bone marrow AT | 1.2 ± 0.4 | 1.8 ± 0.8 | .10 |
| Brown AT | 5.1 ± 2.4 | 4.8 ± 1.6 | .48 |

EGP endogenous glucose production, R_d_ rate of disappearance, AT adipose tissue. The data are means and SDs, and the *P* values are from *t* test comparisons between groups.
